# Comparative effectiveness and safety between oxaliplatin-based and cisplatin-based therapy in advanced gastric cancer: A meta-analysis of randomized controlled trials

**DOI:** 10.18632/oncotarget.9189

**Published:** 2016-05-05

**Authors:** Jun Huang, Yongzhao Zhao, Yong Xu, Yanjie Zhu, Jiale Huang, Yanna Liu, Liying Zhao, Zhijia Li, Hao Liu, Qi-long Wang, Xiaolong Qi

**Affiliations:** ^1^ Department of Gastrointestinal Surgery, The Second Affiliated Hospital of Nanchang University, Nanchang, China; ^2^ School of Medicine, Tongji University, Shanghai, China; ^3^ Department of Nephrology, The Affiliated Huai'an Hospital of Xuzhou Medical College and The Second People's Hospital of Huai'an, Huai'an, China; ^4^ Department of General Surgery, Nanfang Hospital, Southern Medical University, Guangzhou, China; ^5^ Department of Clinical Oncology, Huai'an First People's Hospital, Nanjing Medical University, Huai'an, China

**Keywords:** effectiveness, safety, chemotherapy, advanced gastric cancer

## Abstract

**Background & Aims:**

Platinum-based drugs are the most significant chemotherapy for advanced gastric cancer. The study aims to compare the efficacy and safety of oxaliplatin-based therapy versus cisplatin-based therapy in patients with advanced gastric cancer.

**Materials and Methods:**

An adequate literature search in EMBASE, Cochrane Central Register of Controlled Trials (CENTRAL), MEDLINE, American Society of Clinical Oncology (ASCO) and European Society of Medical Oncology (ESMO) was conducted. Phase II or III randomized controlled trials (RCTs) that compared effectiveness and safety between oxaliplatin-based and cisplatin-based therapy in patients with advanced gastric cancer were eligible. The primary endpoint was overall response rate (ORR), progression free survival (PFS) and overall survival (OS). The second endpoint was the adverse events.

**Results:**

Five phase II or III RCTs involving a total of 2,046 patients were identified. The results showed that there were no significant difference in ORR (OR = 1.17, 95% CI = 0.98–1.40, *p* = 0.08, I^2^ = 0%), PFS (HR = 0.92, 95% CI = 0.84–1.01, *p* = 0.09, I^2^ = 0%) and OS (HR = 0.91, 95% CI = 0.82–1.01, *p* = 0.07, I^2^ = 0%) between oxaliplatin-based therapy and cisplatin-based therapy. In addition, oxaliplatin-based therapy had lower risk of neutropenia, anemia, nausea, alopecia, thromboembolism, stomatitis and creatinine increased at all grades, and neutropenia, anemia, leukopenia and alopecia at 3–4 grades than cisplatin-based therapy. However, oxaliplatin-based therapy was associated with increased risk of neurosensory toxicity and thrombocytopenia.

**Conclusions:**

Our meta-analysis showed that there were no significant difference in ORR, PFS and OS between oxaliplatin-based therapy and cisplatin-based therapy. The oxaliplatin-based therapy could generally decrease the risk of adverse effects except neurosensory toxicity and thrombocytopenia.

## INTRODUCTION

Around one million people are newly diagnosed with gastric cancer all over the world every year. The American Cancer Society estimates there would be 24,590 new gastric cancer cases and 10,720 gastric cancer deaths in 2015 in the United States [1]. Currently, the curative surgical resection is the mainstay of treatment for gastric cancer. However, approximately two thirds of the patients miss the chance of radical surgery and fall on the list of palliative chemotherapy with a relative disappointing outcome [2].

Platinum-based drugs are generally adopted as anticancer therapies for various cancers by binding to DNA strands of tumor cells and interfering with its replication [3]. As a platinum drug, cisplatin plays an important role in the treatment of advanced gastric cancer. Patients with advanced gastric cancer were treated with S-1 plus cisplatin as the first-line treatment presenting markedly longer median overall survival (OS) and progression-free survival (PFS) than those treated with S-1 alone [4]. In addition, irinotecan could significantly prolong the PFS when combined with cisplatin than it alone [5]. Oxaliplatin, a new promising anticancer drug, also has obviously inhibitive effect on locally advanced or metastatic gastric cancer [6]. A randomized, open-label, multicenter phase III study presented that oxaliplatin plus S-1 was as effective as cisplatin plus S-1 and with favorable safety profile in the palliative chemotherapy for advanced gastric cancer [7].

*Montagnani et al.* performed a meta-analysis to compare the effectiveness and safety profile between the cisplatin-based and oxaliplatin-based palliative chemotherapy for advanced and unresectable gastric cancer [8]. The results demonstrated that oxaliplatin apparently improved the PFS and OS [8]. However, a randomized phase II study reported that there were no significant difference between oxaliplatin-based therapy and cisplatin-based therapy for advanced gastric cancer in terms of overall response rate (ORR), PFS and OS [9]. Another randomized phase III study also proved that there was no statistical difference between the two strategies in terms of PFS and OS [7]. Therefore, the aim of this study was to review the available literature and perform an updated meta-analysis of comparative effectiveness and safety between oxaliplatin-based and cisplatin-based therapy in the treatment of advanced gastric cancer.

## RESULTS

### Literature search

**Figure 1:**
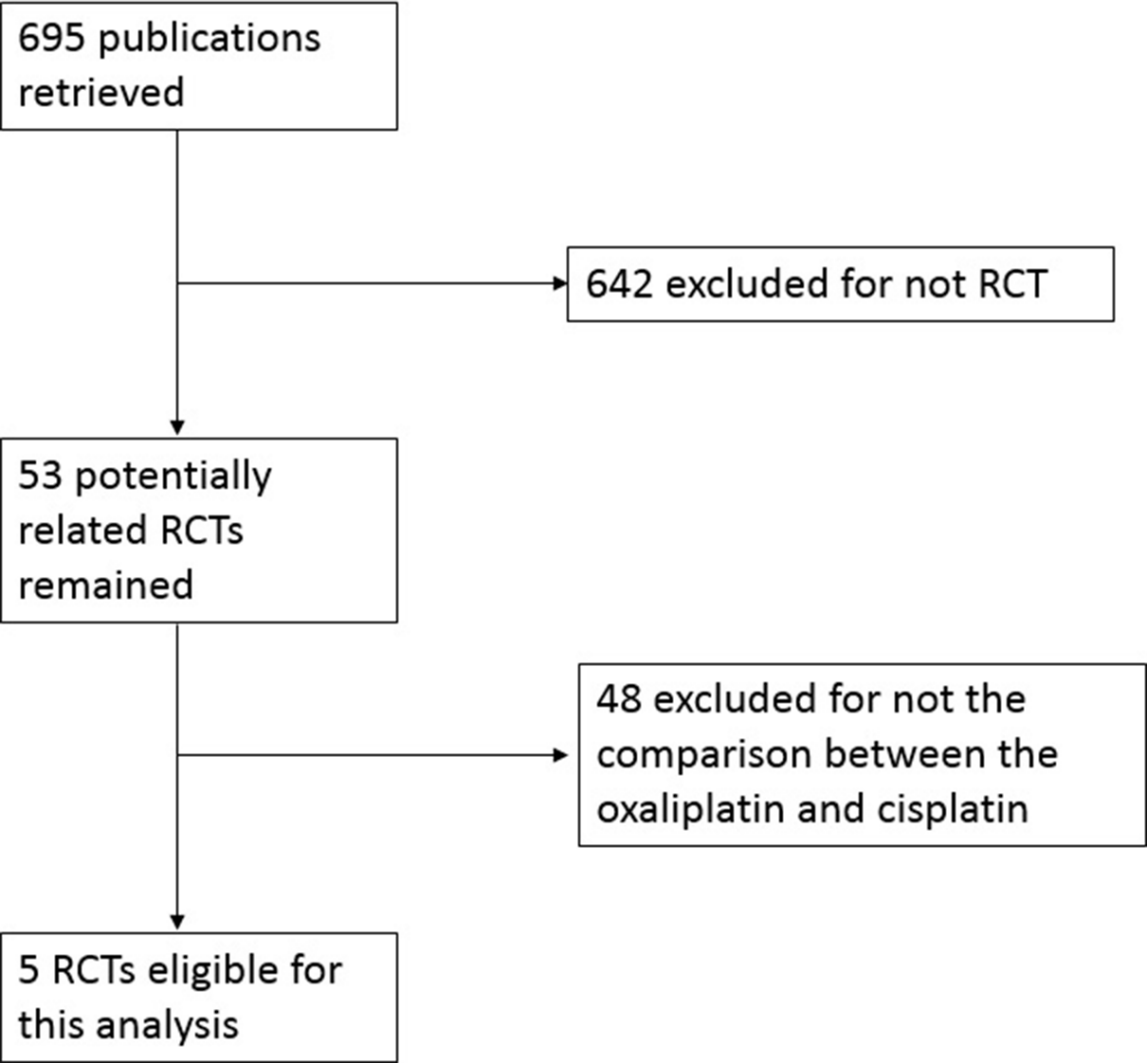
Flow diagram of study selection process

As shown in Figure 1, 695 initial articles including our search terms were obtained. In these studies, 642 were excluded for not randomized controlled trials (RCTs). The rest 53 articles were retrieved for full-text review, from which 48 were further excluded for not the comparison between oxaliplatin-based therapy and cisplatin-based therapy. Finally, 5 studies including 2,046 patients met the inclusion criteria and were included in the meta-analysis [7, 9–12].

### Characteristics of included studies

The detailed characteristics of included studies were listed in Table 1. The median age of patients in included studies ranged from 56 to 65 years old. The five studies were made up of two phase II RCTs and three phase III RCTs. All studies reported the ORR, PFS, OS and toxicities. For toxicities, the study by *Popov et al.* was excluded for reporting the AEs by the cycles of treatment, the other four studies by the numbers of patients were enrolled. Two studies appraised the comparison between oxaliplatin-based and cisplatin-based therapy in two-drug regimen [7, 9] while the others appraised the comparison in three-drug regimen [10–12]. The study by *Cunningham et al.* was two-by-two design and divided into two parts according to containing oxaliplatin or cisplatin, respectively [11].

**Table 1:** Characteristics of the included studies

Study	Year	Clinical trial	Patients (*n*)	Male (%) (Arm-1 vs Arm-2)	Median age (Years)	Treatment	Jadad score
*Yamada et al. [7]*	2015	Phase III	685	75.5% vs 73.1%	65 vs 65	Arm-1: 80–120 mg/day S-1 for 2 weeks with 100 mg/m^2^ oxaliplatin on day 1, every 3 weeksArm-2: S-1 for 3 weeks with 60 mg/m^2^ cisplatin on day 8, every 5 weeks	3
*Kim et al. [9]*	2014	Phase II	77	67.0% vs 74.0%	58 vs 56	Arm-1: 35 mg/m^2^ docetaxel weekly on days 1 and 8 and 120 mg/m^2^ oxaliplatin on day 1, every 3 weeksArm-2: 35 mg/m^2^ docetaxel weekly on days 1 and 8 and 60 mg/m2 on day 1, every 3 weeks	2
*Al-Batran et al. [10]*	2008	Phase III	220	57.1% vs 75.0%	64 vs 64	Arm-1: oxaliplatin 85 mg/m^2^ day 1, 5-FU 2600 mg/m^2^ 24 h-c.i. day 1, FA 200 mg/m^2^ day 1, every 2 weeksArm-2: cisplatin 50 mg/m2 day 1, 5-FU, every 2 weeks	2
*Cunningham et al. (1) [11]*	2008	Phase III	508	81.3% vs 81.1%	61 vs 65	Arm-1: epirubicin 60 mg/m^2^ day 1, oxaliplatin 85 mg/m^2^ day 1, 5-FU c.i. 200 mg/m^2^ daily, every 3 weeksArm-2: epirubicin 60 mg/m^2^ day 1, cisplatin 50 mg/m^2^ day 1, 5-FU c.i. 200 mg/m^2^, every 3 weeks	3
*Cunningham et al. (2) [11]*	2008	Phase III	494	82.8% vs 80.5%	62 vs 64	Arm-1: epirubicin 60 mg/m^2^ day 1, oxaliplatin 85 mg/m^2^ day 1, capecitabine 625 mg/m^2^ × 2 daily, every 3 weeksArm-2: epirubicin 50 mg/m^2^ day 1, cisplatin 50 mg/m^2^ day 1, capecitabine 625 mg/m^2^ × 2 daily, every 3 weeks	3
*Popov et al. [12]*	2008	Phase II	62	66.7% vs 72.2%	57 vs 55	Arm-1: oxaliplatin 85 mg/m^2^ day 1, 5-FU bolus 400 mg/m^2^ day 1, 2, 5-FU 600 mg/m2 22 h c.i. day 1, 2, FA = 200 mg/m^2^ d1, 2, every 2 weeksArm-2: cisplatin 50 mg/m^2^ day 1,5-FU bolus 400 mg/m^2^ day 1, 2, 5-FU 600 mg/m^2^ 22 h c.i. day 1, 2, FA = 200 mg/m^2^ day 1, 2, every 2 weeks	2

c.i. Continuous infusion; 5-FU 5-fluorouracil; FA folinic acid.

### Meta-analyses of ORR

**Figure 2:**
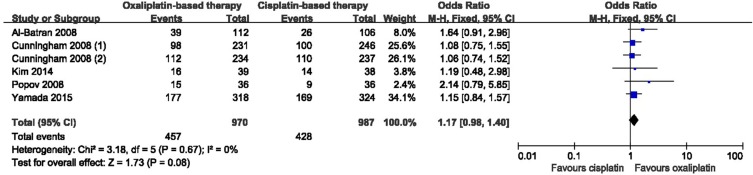
Meta-analysis of overall response rate

Five eligible studies all covered the ORR. As shown in Figure 2, the results of the meta-analysis presented that there were no significant difference between oxaliplatin-based and cisplatin-based therapy, and no heterogeneity among the studies (OR = 1.17, 95% Confidence Intervals (CI) = 0.98–1.40, *p* = 0.08, I^2 =^ 0%). In addition, there was no bias among all included studies (Begg test, *p* = 0.133; Egger test, *p* = 0.099), and no decisive effect according to the influence analysis conducted by stata12.0 (Supplementary Figure 1).

### Meta-analyses of PFS

**Figure 3:**
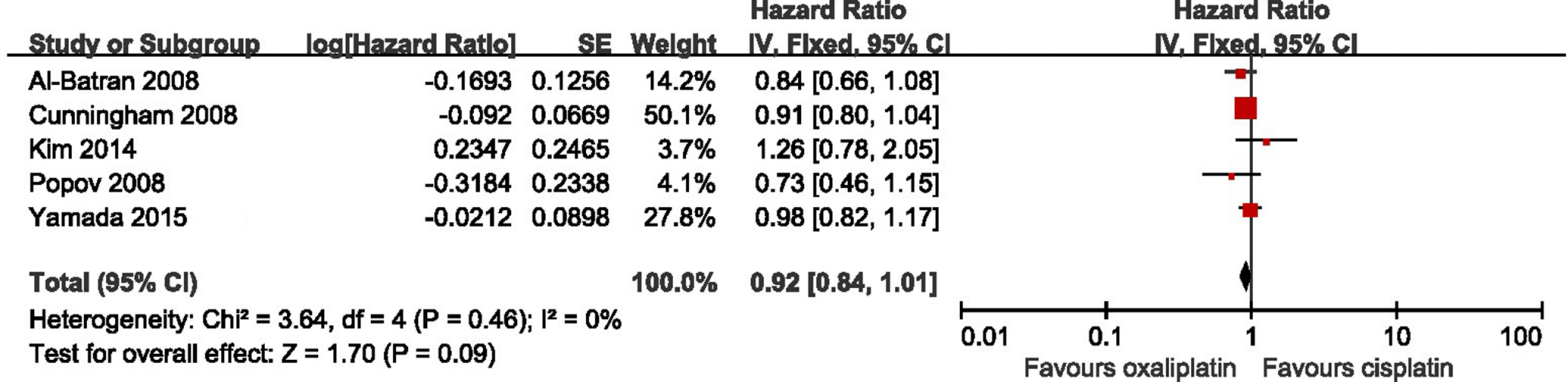
Meta-analysis of progression free survival

Five eligible studies all reported the PFS. There was no significant heterogeneity between these studies when pooling the HR, so HR was pooled in the fixed model. As shown in Figure 3, the results presented 8% improvement of PFS in the oxaliplatin-based therapy, but with no statistically significant (HR = 0.92, 95% CI = 0.84–1.01, *p* = 0.09, I^2^ = 0%). Besides, there was no bias among all included studies from the Begg test and Egger test (Begg test, *p* = 1.000; Egger test, *p* = 0.963), and no decisive effect according to the influence analysis (Supplementary Figure 2).

### Meta-analyses of OS

**Figure 4:**
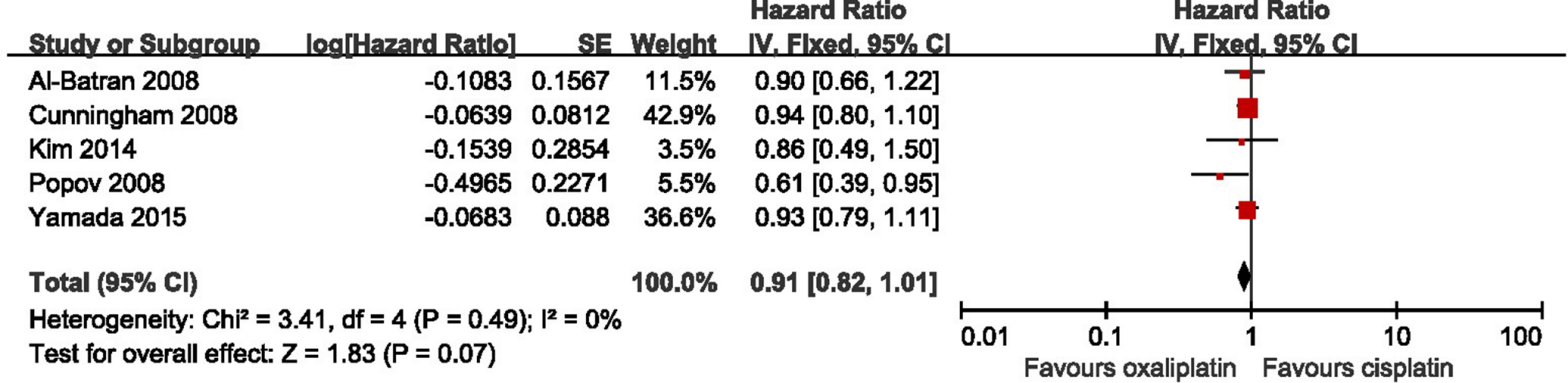
Meta-analysis of overall survival

The OS was also reported in five studies. As shown in Figure 4, a fixed model was used to pool HR because there is no significant heterogeneity among included studies. The results indicated a slight improvement of OS in oxaliplatin-based therapy group without statistically significant (HR = 0.91, 95% CI = 0.82–1.01, *p* = 0.07, I^2^ = 0%). No bias among all included studies was detected, (Begg test, *p* = 0.086; Egger test, *p* = 0.174), and the influence analysis showed no conclusive effect (Supplementary Figure 3).

### Meta-analyses of AEs

All-grade AEs were listed in Table 2. The oxaliplatin-based therapy could significantly decrease the risk of neutropenia (OR = 0.63, 95% CI = 0.40–0.99, *p* = 0.04), anemia (OR = 0.50, 95% CI = 0.41–0.61, *p* < 0.0001), nausea (OR = 0.65, 95% CI = 0.50–0.86, *p* = 0.003), stomatitis (OR = 0.79, 95% CI = 0.66–0.96, *p* = 0.02), increasing of creatinine (OR = 0.24, 95% CI = 0.07– 0.77, *p* = 0.02) and thromboembolism (OR = 0.42, 95% CI = 0.28–0.64, *p* < 0.0001). However, the oxaliplatin-based therapy markedly increased the risk of neurosensory toxicity (OR = 8.68, 95% CI = 5.28–14.27, *p* < 0.0001) and thrombocytopenia (OR = 1.29, 95% CI = 1.04–1.61,*p* = 0.02) compared to the cisplatin-based therapy. There were no statistically significant differences in febrile neutropenia, leukopenia, vomiting, diarrhea, fatigue and alopecia between the two arms.

**Table 2:** Adverse events of the included studies

Adverse events	All grades			Grades 3–4		
	OR (95% CI)	*p* value	I^2^	OR (95% CI)	*p* value	I^2^
Neutropenia [7, 9–11]	0.63 (0.40, 0.99)	0.04‡	76%	0.50 (0.35, 0.71)	0.001‡	62%
Febrile neutropenia [7, 11]	0.55 (0.21, 1.39)	0.21	80%	0.59 (0.24, 1.45)	0.25	73%
Leukopenia [7, 9, 10]	0.69 (0.43, 1.11)	0.12	55%	0.34 (0.15, 0.78)	0.01‡	62%
Thrombocytopenia [7, 9–11]	1.29 (1.04, 1.61)	0.02‡	0%	0.99 (0.69, 1.42)	0.95	0%
Anemia [7, 9–11]	0.50 (0.41, 0.61)	< 0.0001‡	47%	0.47 (0.36, 0.62)	< 0.0001‡	36%
Nausea [7, 10]	0.65 (0.50, 0.86)	0.003‡	30%	0.78 (0.41, 1.47)	0.44	5%
Vomiting [7, 10]	0.66 (0.29, 1.50)	0.32	85%	0.42 (0.14, 1.22)	0.11	0%
Diarrhea [7, 9–12]	0.83 (0.68, 1.01)	0.06	32%	1.25 (0.90, 1.73)	0.19	25%
Stomatitis [7, 9–12]	0.79 (0.66, 0.96)	0.02‡	0%	1.52 (0.80, 2.87)	0.20	0%
Fatigue [7, 9, 10]	0.74 (0.45, 1.24)	0.25	54%	0.93 (0.39, 2.22)	0.87	52%
Alopecia [9–12]	0.68 (0.45, 1.03)	0.07	51%	0.46 (0.35, 0.60)	< 0.0001‡	0%
Neurosensory toxicity [7, 9–12]	8.68 (5.28, 14.27)	< 0.0001‡	78%	8.37 (3.99, 17.59)	< 0.0001‡	32%
Creatinine [7, 9]	0.24 (0.07, 0.77)	0.02‡	68%	0.16 (0.02, 1.36)	0.09	NA
Thromboembolism [10, 11]	0.42 (0.28, 0.64)	< 0.0001‡	10%	NA	NA	NA

CI: confidence interval, NA: not assessable, ‡: *p* < 0.05: the difference is significant.

For the 3–4 grades AEs (Table 2), the risk of neutropenia (OR = 0.50, 95% CI = 0.35–0.71, *p* = 0.001), leukopenia (OR = 0.34, 95% CI = 0.15–0.78, *p* = 0.01), anemia (OR = 0.47, 95% CI = 0.36–0.62, *p* < 0.0001) and alopecia (OR = 0.46, 95% CI = 0.35–0.60, *p* = 0.0001) were obviously lower in the oxaliplatin-based therapy. However, the risk of neurosensory toxicity (OR = 8.37, 95% CI = 3.99–17.59, *p* = 0.01) increased again in oxaliplatin-based therapy. No statistical differences in febrile neutropenia, thrombocytopenia, nausea, diarrhea, stomatitis, fatigue, vomiting, increasing of creatinine was observed between the two therapies.

## DISCUSSION

In recent years, palliative chemotherapy (e.g. fluoropyrimidine, platinum compounds, docetaxel and epirubicin) prolongs the survival and improves the quality of life in patients with metastatic gastric cancer [13]. A recent meta-analysis presented that the platinum-based therapy significantly improved the therapeutic effect compared to non-platinum-based therapy in patients with advanced gastric cancer [14]. As the most common platinum drugs, oxaliplatin and cisplatin are both of great importance in the treatment of advanced gastric cancer. Plenty of clinical trials were conducted to explore the effect of oxaliplatin in advanced gastric cancer [15–20]. In the study *by Lu et al.*, the results showed that oxaliplatin combined with S-1 significantly increased the median survival time (14.0 versus 11.0 months, *p* = 0.03), PFS (6.5 versus 4.0 months, *p* = 0.02), and 1-year survival rate (63.8% versus 48.9%) than S-1 alone [16]. Cisplatin plays also an active role in the chemotherapeutic therapy and studies were well conducted to evaluate the function of cisplatin in the treatment of advanced gastric cancer [4, 16, 21–23]. In the study by *Koizumi et al.*, 148 patients were assigned to S-1 plus cisplatin and 150 patients were assigned to S-1 alone. The results showed that the time of median OS (13.0 vs 11.0 months, *p* = 0.04) and PFS (6.0 vs 4.0 months, *p* < 0.0001) was significantly increased in the group of S-1 plus cisplatin, however, there were more severe (3–4 grades) AEs including leucopenia, neutropenia, anemia, nausea, and anorexia in the combined group.

Based on the better therapeutic effects of platinum-based therapy for advanced gastric cancer, efforts have been made to explore the differences of efficacy and safety between oxaliplatin-based and cisplatin-based therapy [7, 9–11, 24]. The results of a meta-analysis including three RCTs indicated that oxaliplatin-based therapy significantly improved PFS and OS than cisplatin-based therapy. Moreover, the oxaliplatin-based therapy was also associated with less neutropenia and fewer thromboembolic events, but with increased neurotoxicity [8].

In our meta-analysis, compared with the previous meta-analysis by *Montagnani et al.*, two more RCTs conducted by *Yamada et al.* and *Kim et al.* were enrolled, and both of them supported the results that no significant differences in terms of PFS and OS were observed between oxaliplatin-based and cisplatin-based therapy [7, 9]. Therefore, in contrast with the study by *Montagnani et al.*, our results revealed that there were no significant differences in ORR, PFS and OS between oxaliplatin-based and cisplatin-based therapy. Besides, *Gong et al.* reported that oxaliplatin-based therapy was superior to cisplatin-based therapy. However, the major enrolled studies were retrospective and not RCTs [25]. Another study by *Hamada et al.* focused on the combination of S-1 and was the indirect comparison between oxaliplatin-based and cisplatin-based therapy [26]. Therefore, the results of our study were more convincing and reliable. In addition, the study conducted by *Al-Batran et al.* showed that oxaliplatin-based therapy improved the ORR, PFS and OS compared to the cisplatin-based therapy, which remained us to pay close attention to the age of population for receiving the oxaliplatin-based therapy [10]. Oxaliplatin-based therapy had lower risk of neutropenia, anemia, nausea, alopecia, thromboembolism, stomatitis and creatinine increased at all grades, and neutropenia, anemia, leukopenia and alopecia at 3–4 grades than cisplatin-based therapy. However, oxaliplatin-based therapy was associated with increased risk of neurosensory toxicity and thrombocytopenia. The results in our meta-analysis were different with the study conducted by *Montagnani et al.* in terms of PFS and OS [8]. In addition, no significant differences were found in thrombocytopenia and stomatitis at all grades, alopecia and anemia at 3–4 grades in the previous meta-analysis. However, a significant difference was observed in terms of diarrhea at 3–4 grades in the previous meta-analysis [8]. In our study, more AEs (e.g. fatigue, creatinine increased) were reported and lower risks of thrombocytopenia and stomatitis were detected in oxaliplatin-based therapy. Hence, more efforts should be made to explore the strengths and weaknesses of oxaliplatin-based therapy compared to cisplatin-based therapy.

The highlighted strength of our meta-analysis was that five RCTs with 2,046 patients were included compared with three RCTs with 1294 patients in study by *Montagnani et al.* A updated analysis with a larger population was more convincing. Nonetheless, our meta-analysis is not without limitations. First, the HR of PFS or OS could not be obtained directly from included studies [9, 10, 12]. Besides, few studies were included to analyze certain AEs (e.g. creatinine), which influenced the reliability of the results. Finally, the effects of confounding factors (e.g. sex, drug dosage) were not analyzed for half-baked data.

In conclusion, oxaliplatin-based therapy was not significantly superior to cisplatin-based therapy in terms of ORR, PFS and OS for advanced gastric cancer. Oxaliplatin-based therapy reduced the risk of most AEs, but with a higher occurrence of neurosensory toxicity and thrombocytopenia.

## MATERIALS AND METHODS

### Literature search strategy

Cochrane Controlled Trials Registry, PubMed and EMBASE database were searched up to April 10, 2016. We also reviewed the Annual meeting proceedings from European Society of Medical Oncology (ESMO) and American Society of Clinical Oncology (ASCO). The search strategy was “platinum or cisplatin or oxaliplatin” in combination with “gastric cancer or gastric carcinoma or stomach neoplasm or gastro-oesophageal cancer or oesophago-gastric cancer”, “metastatic or advanced or unresectable or recurrent or stage IV” and “RCTs or randomized controlled trials”. All eligible studies were retrieved, and their reference lists were checked for additional relevant publications.

### Inclusion criteria

Studies researching comparisons of effectiveness and safety profile between oxaliplatin-based and cisplatin-based therapy in patients with advanced gastric cancer were eligible for inclusion. Studies that met all the following criteria were included: (i) English articles; (ii) reporting effectiveness and safety profile of oxaliplatin-based and cisplatin-based therapy in patients with advanced gastric cancer; (iii) studies design of phase II/III randomized controlled trials (iv) enough data to calculate odds ratio (OR) and hazard ratio (HR).

### Exclusion criteria

The exclusion criteria were as follows: (i) not phase II/III randomized controlled trials; (ii) ongoing studies; (iii) review articles; (iv) studies not within the field of interest of this study.

### Data extraction

As for each study, the following information was extracted: year of publication, trial phase, the first author's surname, number of subjects, the percentage of male, median age, treatment arm, effectiveness and frequency of all-grade and severe adverse events (AEs) assessed by the National Cancer Institute Common Toxicity Criteria (CTC) in each arm. Data extraction and information on study design, outcomes were performed by two independent reviewers (Zhao Y and Huang J) and disagreements were resolved by discussion and consensus with a third reviewer (Qi X).

### Statistical analysis

Effect estimates were analyzed with Review Manager 5.2. Dichotomous data were compared using an OR. The HRs with their 95% CIs were directly obtained from the article or calculated by using previously published methods [27]. Forest plots were generated for graphical presentations, and heterogeneity among different studies was appraised by Q statistics and I^2^ estimates. Fixed-effects model was used to aggregate data if there were no statistical heterogeneity. However, when effects were heterogeneous (I^2^ > 50%), randomized effects model was carried out. Publication bias was examined using analyses described by Egger and Begg by stata12.0. Influence analysis was employed to the study by stata12.0. The 95% CI for each result were computed.

## SUPPLEMENTARY MATERIALS FIGURES



## References

[1] Siegel RL, Miller KD, Jemal A (2015). Cancer statistics, 2015. CA Cancer J Clin.

[2] Pozzo C, Barone C (2008). Is there an optimal chemotherapy regimen for the treatment of advanced gastric cancer that will provide a platform for the introduction of new biological agents?. Oncologist.

[3] Yasui H, Tsurita G, Imai K (2014). DNA synthesis inhibitors for the treatment of gastrointestinal cancer. Expert Opin Pharmacother.

[4] Koizumi W, Narahara H, Hara T, Takagane A, Akiya T, Takagi M, Miyashita K, Nishizaki T, Kobayashi O, Takiyama W, Toh Y, Nagaie T, Takagi S (2008). S-1 plus cisplatin versus S-1 alone for first-line treatment of advanced gastric cancer (SPIRITS trial): a phase III trial. Lancet Oncol.

[5] Higuchi K, Tanabe S, Shimada K, Hosaka H, Sasaki E, Nakayama N, Takeda Y, Moriwaki T, Amagai K, Sekikawa T, Sakuyama T, Kanda T, Sasaki T (2014). Biweekly irinotecan plus cisplatin versus irinotecan alone as second-line treatment for advanced gastric cancer: a randomised phase III trial (TCOG GI-0801/BIRIP trial). Eur J Cancer.

[6] Louvet C, André T, Tigaud JM, Gamelin E, Douillard JY, Brunet R, François E, Jacob JH, Levoir D, Taamma A, Rougier P, Cvitkovic E, de Gramont A (2002). Phase II study of oxaliplatin, fluorouracil, and folinic acid in locally advanced or metastatic gastric cancer patients. J Clin Oncol.

[7] Yamada Y, Higuchi K, Nishikawa K, Gotoh M, Fuse N, Sugimoto N, Nishina T, Amagai K, Chin K, Niwa Y, Tsuji A, Imamura H, Tsuda M (2015). Phase III study comparing oxaliplatin plus S-1 with cisplatin plus S-1 in chemotherapy-naive patients with advanced gastric cancer. Ann Oncol.

[8] Montagnani F, Turrisi G, Marinozzi C, Aliberti C, Fiorentini G (2011). Effectiveness and safety of oxaliplatin compared to cisplatin for advanced, unresectable gastric cancer: a systematic review and meta-analysis. Gastric Cancer.

[9] Kim YS, Sym SJ, Park SH, Park I, Hong J, Ahn HK, Park J, Cho EK, Lee WK, Chung M, Lee JH, Shin DB (2014). A randomized phase II study of weekly docetaxel/cisplatin versus weekly docetaxel/oxaliplatin as first-line therapy for patients with advanced gastric cancer. Cancer Chemother Pharmacol.

[10] Al-Batran SE, Hartmann JT, Probst S, Schmalenberg H, Hollerbach S, Hofheinz R, Rethwisch V, Seipelt G, Homann N, Wilhelm G, Schuch G, Stoehlmacher J, Derigs HG (2008). Phase III trial in metastatic gastroesophageal adenocarcinoma with fluorouracil, leucovorin plus either oxaliplatin or cisplatin: a study of the Arbeitsgemeinschaft Internistische Onkologie. J Clin Oncol.

[11] Cunningham D, Starling N, Rao S, Iveson T, Nicolson M, Coxon F, Middleton G, Daniel F, Oates J, Norman AR (2008). Upper Gastrointestinal Clinical Studies Group of the National Cancer Research Institute of the United Kingdom Capecitabine and oxaliplatin for advanced esophagogastric cancer. N Engl J Med.

[12] Popov I, Radosevic-Jelic L, Jezdic S, Milovic M, Borojevic N, Stojanovic S, Stankovic V, Josifovski T, Kezic I (2008). Biweekly oxaliplatin, fluorouracil and leucovorin versus cisplatin, fluorouracil and leucovorin in patients with advanced gastric cancer. Journal of BUON.

[13] Bilici A (2014). Treatment options in patients with metastatic gastric cancer: current status and future perspectives. World J Gastroenterol.

[14] Chen WW, Wang F, Xu RH (2013). Platinum-based versus non-platinum-based chemotherapy as first line treatment of inoperable, advanced gastric adenocarcinoma: a meta-analysis. PLoS One.

[15] Lu Y, Liu Z, Zhang J (2014). S-1 plus oxaliplatin vs. S-1 as first-line treatment in patients with previously untreated advanced gastric cancer: a randomized phase II study. J Chemother.

[16] Oh SY, Kwon HC, Jeong SH, Joo YT, Lee YJ, Cho Sh, Kang MH, Go SI, Lee GW, Kim Hg, Kang JH (2012). A phase II study of S-1 and oxaliplatin (SOx) combination chemotherapy as a first-line therapy for patients with advanced gastric cancer. Invest New Drugs.

[17] Chen H, Guan R, Lei Y, Chen J, Ge Q, Zhang X, Dou R, Chen H, Liu H, Qi X, Zhou X, Chen C (2015). Lymphangiogenesis in gastric cancer regulated through Akt/mTOR-VEGF-C/VEGF-D axis. BMC Cancer.

[18] Kim JY, Ryoo HM, Bae SH, Kang BW, Chae YS, Yoon S, Baek JH, Kim MK, Lee KH, Lee SA, Song HS, Kim JG (2015). Multi-center Randomized Phase II Study of Weekly Docetaxel Versus Weekly Docetaxel-plus-Oxaliplatin as a Second-line Chemotherapy for Patients with Advanced Gastric Cancer. Anticancer Res.

[19] Chao Y, Hsieh JS, Yeh HT, Su YC, Wu CC, Chen JS, Tai CJ, Bai LY, Yeh KH, Su WC, Li CP (2014). A multicenter phase II study of biweekly capecitabine in combination with oxaliplatin as first-line chemotherapy in patients with locally advanced or metastatic gastric cancer. Cancer Chemother Pharmacol.

[20] Bando H, Yamada Y, Tanabe S, Nishikawa K, Gotoh M, Sugimoto N, Nishina T, Amagai K, Chin K, Niwa Y, Tsuji A, Imamura H, Tsuda M (2015). Efficacy and safety of S-1 and oxaliplatin combination therapy in elderly patients with advanced gastric cancer. Gastric Cance.

[21] Koizumi W, Tanabe S, Saigenji K, Ohtsu A, Boku N, Nagashima F, Shirao K, Matsumura Y, Gotoh M (2003). Phase I/II study of S-1 combined with cisplatin in patients with advanced gastric cancer. Br J Cancer.

[22] Qi X, Zhang L, Lu X (2016). New insights into epithelial-to-mesenchymal transition in cancer. Trends Pharmacol Sci.

[23] Chua C, Tan IB, Yamada Y, Rha SY, Yong WP, Ong WS, Tham CK, Ng M, Tai DW, Iwasa S, Lim HY, Choo SP (2015). Phase II study of trastuzumab in combination with S-1 and cisplatin in the first-line treatment of human epidermal growth factor receptor HER2-positive advanced gastric cancer. Cancer Chemother Pharmacol.

[24] Sumpter K, Harper-Wynne C, Cunningham D, Rao S, Tebbutt N, Norman AR, Ward C, Iveson T, Nicolson M, Hickish T, Hill M, Oates J (2005). Report of two protocol planned interim analyses in a randomised multicentre phase III study comparing capecitabine with fluorouracil and oxaliplatin with cisplatin in patients with advanced oesophagogastric cancer receiving ECF. Br J Cancer.

[25] Gong JF, Shen L (2009). [Meta-analyses of oxaliplatin-based chemotherapy versus cisplatin-based chemotherapy in advanced gastric cancer]. [Article in Chinese]. Zhonghua yi xue za zhi.

[26] Hamada C, Yamada Y, Azuma M, Nishikawa K, Gotoh M, Bando H, Sugimoto N, Nishina T, Amagai K, Chin K, Niwa Y, Tsuji A, Imamura H (2016). Meta-analysis supporting noninferiority of oxaliplatin plus S-1 to cisplatin plus S-1 in first-line treatment of advanced gastric cancer (G-SOX study): indirect comparison with S-1 alone. Int J Clin Oncol.

[27] Tierney JF, Stewart LA, Ghersi D, Burdett S, Sydes MR (2007). Practical methods for incorporating summary time-to-event data into meta-analysis. Trials.

