# Supplementary material for: Comparative effectiveness and safety between oxaliplatin-based and cisplatin-based therapy in advanced gastric cancer: A meta-analysis of randomized controlled trials
Source: Oncotarget. 2016 May 5;7(23):34824–31. doi: 10.18632/oncotarget.9189 (PMC5085192; doi:10.18632/oncotarget.9189)
Supplement: Supplementary file 1 [file oncotarget-07-34824-s001.pdf]

Comparative effectiveness and safety between oxaliplatin-based and cisplatin-based therapy in advanced gastric cancer: A meta-analysis of randomized controlled trials

Supplementary Materials

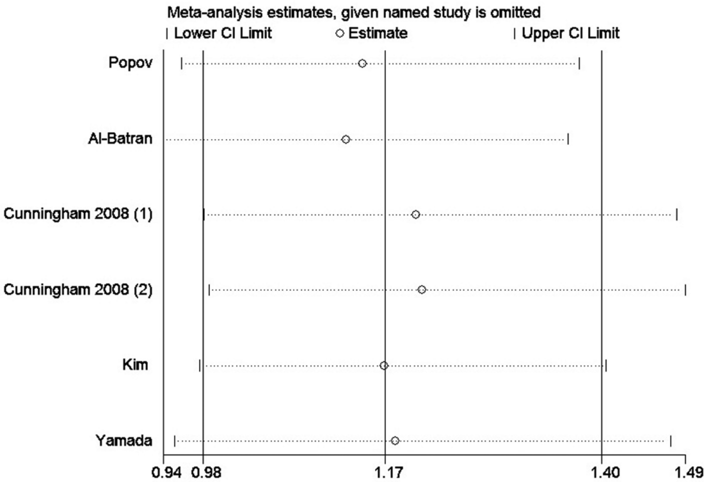

Supplementary Figure S1: Influence analysis of overall response rate.

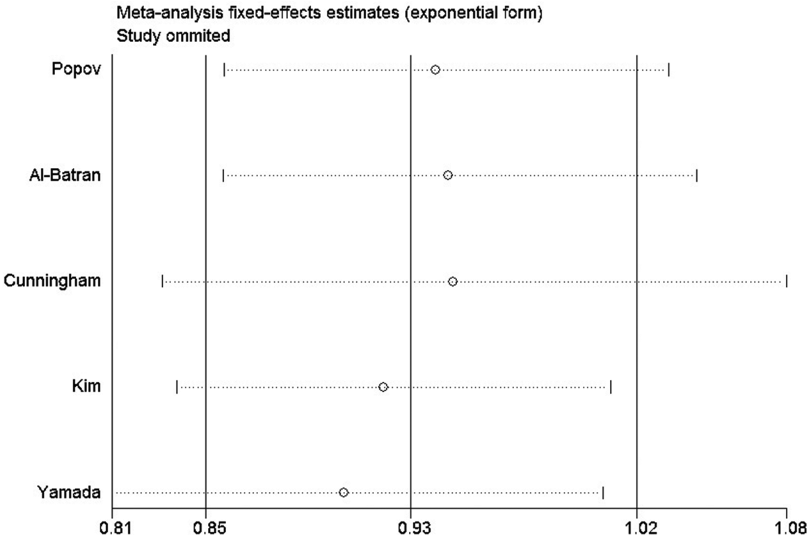

Supplementary Figure S2: Influence analysis of progression free survival.

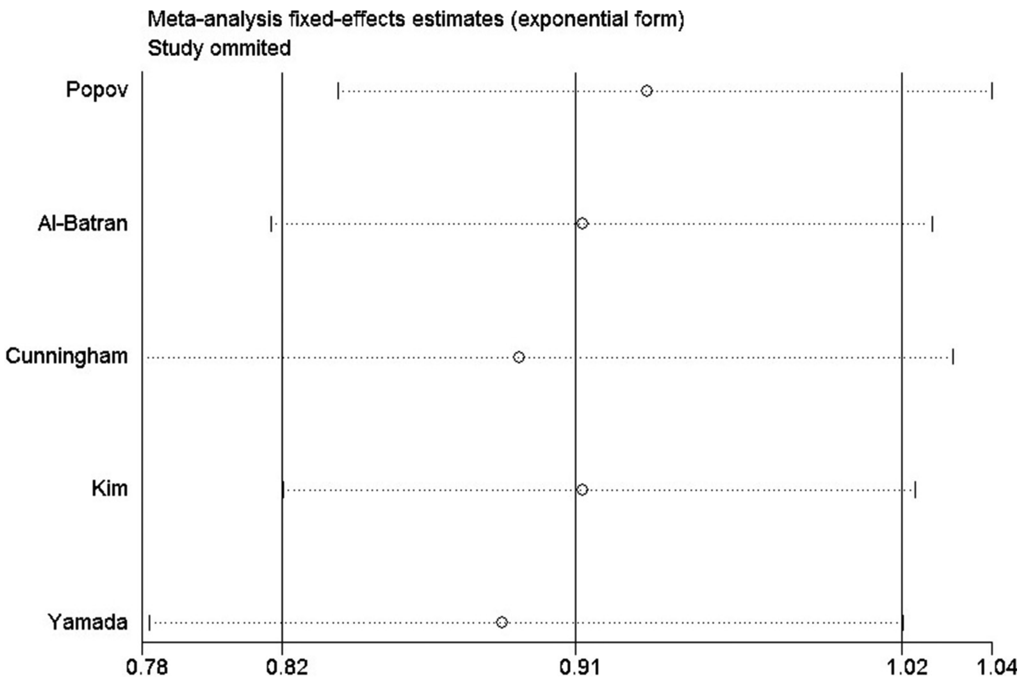

Supplementary Figure S3: Influence analysis of overall survival.
